# Outpatient comprehensive geriatric assessment: effects on frailty and mortality in old people with multimorbidity and high health care utilization

**DOI:** 10.1007/s40520-018-1004-z

**Published:** 2018-07-23

**Authors:** Amelie Lindh Mazya, Peter Garvin, Anne W. Ekdahl

**Affiliations:** 1 Division of Clinical Geriatrics, Department of Neurobiology, Care Sciences and Society (NVS), Karolinska Institutet, Floor 7, 141 83 Huddinge, Sweden; 2 Geriatric Department of Danderyd Hospital, Stockholm, Sweden Danderydsgeriatriken, Mörbygårdsvägen, 182 87 Danderyd, Sweden; 3 Unit of Research and Development in Local Health Care, Region of Östergötland, Linköping, Sweden; 4 Institution of Clinical Research, Helsingborg Hospital, Lund University, Lund, Sweden

**Keywords:** Comprehensive Geriatric Assessment, Outpatient, Frailty, Multimorbidity, Randomized controlled trial, Community dwelling

## Abstract

**Background:**

Multimorbidity and frailty are often associated and Comprehensive Geriatric Assessment (CGA) is considered the gold standard of care for these patients.

**Aims:**

This study aimed to evaluate the effect of outpatient Comprehensive Geriatric Assessment (CGA) on frailty in community-dwelling older people with multimorbidity and high health care utilization.

**Methods:**

The Ambulatory Geriatric Assessment—Frailty Intervention Trial (AGe-FIT) was a randomized controlled trial (intervention group, *n* = 208, control group *n* = 174) with a follow-up period of 24 months. Frailty was a secondary outcome. Inclusion criteria were: age ≥ 75 years, ≥ 3 current diagnoses per ICD-10, and ≥ 3 inpatient admissions during 12 months prior to study inclusion. The intervention group received CGA-based care and tailored interventions by a multidisciplinary team in an Ambulatory Geriatric Unit, in addition to usual care. The control group received usual care. Frailty was measured with the Cardiovascular Health Study (CHS) criteria. At 24 months, frail and deceased participants were combined in the analysis.

**Results:**

Ninety percent of the population were frail or pre-frail at baseline. After 24 months, there was a significant smaller proportion of frail and deceased (*p* = 0.002) and a significant higher proportion of pre-frail patients in the intervention group (*p* = 0.004). Mortality was high, 18% in the intervention group and 26% in the control group.

**Conclusion:**

Outpatient CGA may delay the progression of frailty and may contribute to the improvement of frail patients in older persons with multimorbidity.

## Introduction

Frailty is associated with functional decline, adverse health outcomes, and mortality. Ten percent of all community-dwelling older people are frail, and the prevalence increases with age, affecting one-fourth of the oldest old. The prevention and treatment of frailty thus pose a great challenge to future healthcare systems [1–3]. Multimorbidity is a state with co-occurrence of multiple chronic diseases in the same person. Earlier studies often defines the state by the presence of two or more long-term conditions [4]. Multimorbidity affects 70% of people older than 80 years, and as reported, 46% of frail persons [5, 6]. Many of the geriatric patients are however affected by more than two chronic diseases at the same time. An often used definition of older persons with the highest health care utilization in Sweden consists of an age criteria; 75 years or older, a multimorbidity criteria; three or more concomitant diseases, and a hospitalization criteria; three or more hospitalizations the previous year. This definition is often used by the Swedish National Board of Health and Welfare to describe the top 4% of people aged 75 years or older that have the most complex needs of care [7]. This definition demarcates a population with a high level of multimorbidity and can easily be found in care databases, making it a useful definition of old people with multimorbidity to use in research aiming to evaluate interventions for this at-risk population. Frail older people and those with multimorbidity are at high risk of several negative health outcomes and require person-centered care that addresses their individual needs. The holistic approach of comprehensive geriatric assessment (CGA) suits the need of older at-risk patients, and its effectiveness is well-known [8–10]. CGA-based care results in improved function, decreased institutionalization, and less mortality in aged hospital inpatients [8, 9]. Few studies, however, have evaluated CGA or similar multidisciplinary interventions in outpatient settings. These kind of interventions can possibly slow functional decline, reduce disability, and improve mobility in pre-frail and frail individuals [11–13]. Knowledge of the effects of outpatient CGA on frailty in community-dwelling older individuals with a high level of multimorbidity is, however, limited.

The Ambulatory Geriatric Evaluation: a Frailty Intervention Trail (AGe-FIT) studied the effects of CGA-based, tailored care to older, at-risk individuals in an outpatient setting. It showed that such care, after 3 years had prolonged survival and reduced the number of days in hospital and was considered reasonably cost-effective [14, 15]. The aim of the present study was to analyze the effect of outpatient CGA on frailty among community-dwelling older people with multimorbidity and high health care utilization, compared with usual care, over a 2-year period.

## Methods

### Study design and setting

The AGe-FIT was a prospective, randomized, controlled and assessor-blinded, single-center trial involving two parallel groups. Data collection took place between February 2011 and December 2013. Participants were enrolled continuously during the whole year of 2011. Outcomes were assessed at baseline, 12 and 24 months after inclusion date. The study was conducted in Norrköping, a municipality in southern Sweden with 130,000 residents. In 2010, about 8% of residents were aged ≥ 75 years. The county council of Östergötland provided tax-funded healthcare to all residents at 10 primary care centers and a hospital with 300 beds and 24-h admittance for emergencies. The Geriatric Department at the hospital had a memory clinic and an orthogeriatric ward. The study protocol and results on the primary outcome (number of hospitalizations), other health-related outcomes and cost effectiveness have previously been published [14–17].

### Participants and randomization

Community-dwelling persons aged ≥ 75 years, who had been hospitalized three or more times in the previous year, and had three or more current medical diagnoses according to the International Classification of Diseases, 10th Revision (ICD-10), were eligible for study participation. Potential participants (*n* = 837) were identified using a population-based administrative database maintained by the county council. Randomization to the intervention group (IG) and control group (CG) was performed using a random list generated by SPSS software (PASW Statistics 18; IBM Corporation, Chicago, IL, USA). A total of 382 (45%) participants were included in the IG (*n* = 208) and CG (*n* = 174).

### Frailty assessments

Assessments at baseline and 24 months were conducted by blinded research nurses or an occupational therapist, not involved in the care of study participants. Frailty was measured using the criteria from the Cardiovascular Health Study (CHS): weight loss, weakness, exhaustion, slowness, and low activity level [18]. Measurements for slowness and low activity were measured differently from the original CHS criteria. At baseline, unintentional weight loss was assessed by participants’ estimation of weight loss in the previous year, and current weight was measured using a portable beam scale. The percentage of weight loss was calculated as estimated weight loss / estimated weight one year previously. At 24 months, current weight was measured and the proportion of weight loss was calculated using the value obtained at 12 months: (weight at 12 months – weight at 24 months)/weight at 12 months. A weight loss of ≥ 5% was the cutoff value for frailty. Weakness was measured in kilograms using a Jamar handheld dynamometer; the greatest value of two attempts with the dominant hand was used. The cutoff values were defined according to the weakest quintile in the study conducted by Fried et al. [18] Exhaustion was assessed by two statements from the Center for Epidemiological Studies Depression Scale (CES-D) (“I felt that everything I did was an effort” and “I could not get going”). Response options ranged from 0 to 3 (0 = rarely or none of the time, 1 = some or little of the time, 2 = moderately or much of the time, 3 = most or almost all the time) [19]. A response of alternative 2 or 3 to either statement (or both statements) fulfilled the frailty criterion. Slowness was evaluated using gait speed measurement over a distance of 4 m. Participants were instructed to walk at their normal pace, with a walking aid if used in daily life, and a time ≥ 5 s was categorized as slow. This cutoff value is recommended by the International Academy for Nutrition and Ageing (IANA) [20]. Participants’ activity levels were assessed using the International Physical Activity Questionnaire–Short Form (IPAQ-SF), a validated self-report instrument that summates the duration (minutes) and frequency (days) of different kinds of activity, leading to the categorization of activity level as high, moderate, or low [21, 22]. A low activity level fulfilled the frailty criterion. Participants were classified as robust (no criterion fulfilled), pre-frail (one to two criteria fulfilled), or frail (three or more criteria fulfilled), regardless of missing data.

### Intervention and control procedures

Participants in the IG received the CGA-based intervention in addition to usual care. CGA-based care was provided at an ambulatory geriatric unit (AGU) opened specially for the AGe-FIT, in a real-life, non-academic setting. Patients were assessed according to a standardized procedure [16]. Initially, a nurse and a social worker went home to each participant and administered a survey of health, functional status and need for social care. A pharmacist collected information on compliance with the use of prescribed and non-prescription drugs by telephone. This information was conveyed to the physician, who consulted patients at their homes or at the AGU as part of the initial CGA-based evaluation. All information gathered was discussed at the following interdisciplinary team meeting; two such meetings were held per week. Decisions regarding interventions were made at these meetings, often involving the need for additional assessments, for example, by a physiotherapist, occupational therapist, and/or dietician. When needed, participants were referred to specialized medical care. Personalized care and follow-up plans were created and revised when required, and all participants were offered annual medical evaluations. The tailored interventions were often of preventive nature, such as reduction of polypharmacy, advice on exercise or diet, provision of adaptive equipment, and increased social support. Participants could reach nurses at the AGU directly by telephone during office hours.

Participants in the control group received medical and social care as usual. Healthcare was provided by the acute care hospital, 10 primary care centers, and at home. In most cases, it was initiated by the patients or their relatives.

### Sample size

Sample size calculation for the AGe-FIT study was based on a two-tailed significance level of 5%, power of 80%, and an expected detectable effect over 24 months of a 20% reduction in primary outcome mean hospital admission rate, from five to four admissions per year. The result of a minimum of 142 subjects per arm was increased to 200, considering an estimated 40% attrition rate over the study period due to death, withdrawal, and relocation to nursing homes.

### Statistical analyses

Pearson’s chi-squared test was used to compare categorical data between the IG and CG. Adjusted residuals (*z* scores) were calculated for each cell in the contingency tables and then converted into *p* values. The independent-samples *t* test was used for comparison of means of continuous variables. The significance level was set to *p* ≤ 0.05 for all analyses. Participants with measurements for frailty at baseline and at 24 months were included in the analysis. At 24 months, frail participants were combined with deceased participants, to minimize the risk of mortality bias and to describe the most negative outcomes. Analyses were also performed where frail and deceased participants were analyzed as separate groups. The analysis was made accordingly to the intention-to-treat (ITT) principle. Data were stored and analyzed using SPSS Statistics 22 (IBM Corporation, Armonk, NY, USA).

## Results

**Fig. 1:**
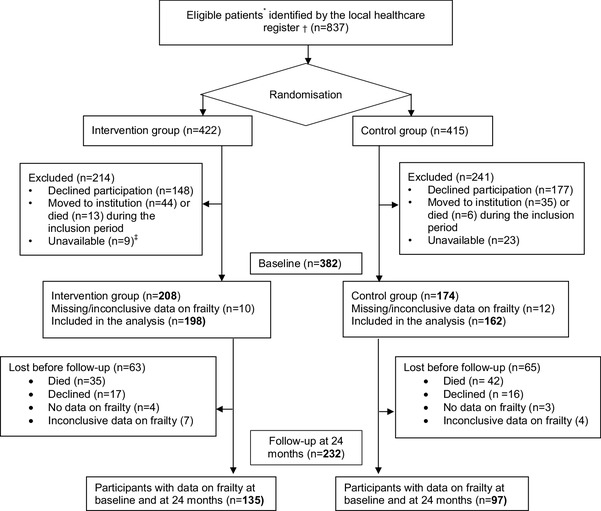
Flowchart of participant selection, frailty assessment and missing data on frailty.*Community dwelling, ≥ 75 years, having received inpatient hospital care ≥ three times during the past 12 months and having ≥ three concomitant medical diagnoses identified from a population-based, administrative database. ^†^Population-based, administrative database run by the County Council. ^‡^After three phone calls, no further attempts to reach the participants in baseline

**Fig. 2:**
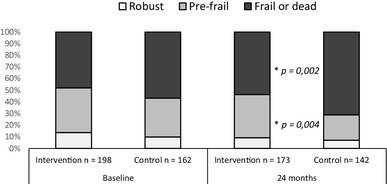
Frailty distributions at baseline and 24 months presented as proportions. At 24 months frail and deceased participants were combined to minimize mortality bias. There was a significant difference between the intervention group and the control group in the distribution of outcomes at 24 months. The proportion of pre-frail participants were larger in the IG (*p* = 0.004) and the proportion of frail and deceased participants were smaller (*p* = 0.002)

**Fig. 3:**
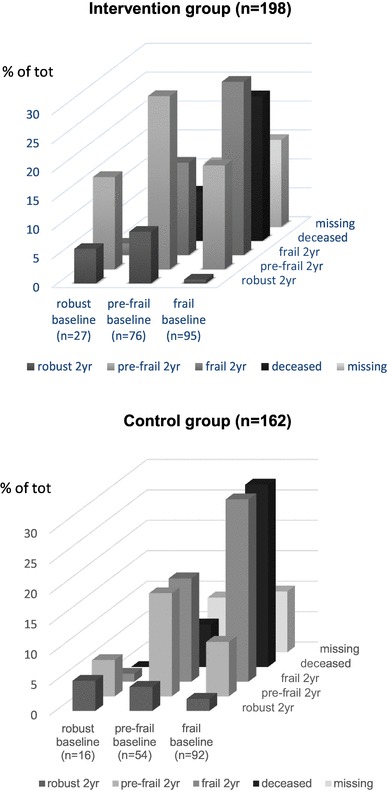
The change in frailty status over 24-months in percent according to baseline status. The figure includes deceased participants and missing data

The mean age of study participants was 82.5 years, and 52% of participants were women. No difference in baseline characteristic was observed between groups (Table 1). Baseline data on frailty were available for 360 (94%) participants (IG, *n* = 198; CG, *n* = 162). Of these, 187 (52%) participants were frail, 130 (36%) were pre-frail, and 43 (12%) participants were robust, with no significant difference between the IG and CG. Eleven participants in the IG did not receive the intervention because they later declined to participate. They were however included in the analysis. Frailty data at the 24-month time point were available for 232 participants (135 in the IG/97 in the CG) (Fig. 1). Of these participants, 108 (47%) were frail, 97 (42%) were pre-frail, and 27 (11%) were robust. At 24 months the IG contained a significantly greater proportion of pre-frail individuals than did the CG (*p* = 0.004; Fig. 2). The proportion of frail and deceased combined was also significantly lower in the IG (*p* = 0.002; Fig. 2). When frail and deceased participants were analyzed as separate groups, there were no significant differences in proportions between the IG and CG (frail, *p* = 0.19, deceased, *p* = 0.051). Mortality rates were high; 35 (18%) participants in the IG and 42 (26%) participants in the CG died, with no significant difference between groups (*p* = 0.051). Very few participants classified as robust at baseline became frail or died. Pre-frail participants mainly stayed pre-frail or became frail. The majority of those classified as frail at baseline, stayed frail or died. More frail participants in the IG improved to pre-frail or robust (*n* = 19, 24%), compared with the CG (*n* = 11, 13%) but this difference in proportions was not statistically significant (*p* = 0.09). The development of frailty status from baseline to 24 months follow-up is illustrated in Fig. 3.

**Table 1:** Baseline basic characteristics of the intervention and control groups

Characteristics	Intervention	Control
No.	208	174
Age (years), mean (SD)	82.3 (4.6)	82.7 (5.1)
Sex (female), *n* (%)	108 (47)	81 (50)
Living alone, *n* (%)	102 (49)	93 (54)
Primary school only, *n* (%)	127 (62)	109 (63)
Hearing impairment with hearing aid, *n* (%)	75 (36)	59 (34)
Vision impairment with glasses, *n* (%)	49 (24)	56 (32)
Mini-Mental State Examination score, mean (SD)	26.2 (3.3)	26.6 (3.0)
Barthel index score, mean (SD)	89.6 (14.8)	92.0.(9.9)

The amounts of missing data were 15.4% (IG) and 16.7% (CG), deceased participants not included. The distribution of categories with missing data was similar in both groups, at baseline and 24 months.

## Discussion

This study showed that outpatient CGA affects frailty in older individuals. At follow-up, the IG contained significantly more pre-frail and less frail/deceased participants than expected. We attribute these results to the effect of the intervention, which was able to delay progression to frailty in pre-frail patients. Our findings support previous studies showing that intervention against frailty is possible, and that CGA, applied in the present study as outpatient care, could be care approach of choice for older at-risk patients [23]. There was also a tendency to improvement of frailty, but not statistically significant in this small sample.

As in other studies of multicomponent interventions, however, which component(s) of the CGA-based intervention had the most impact is unclear. As the interventions were tailored to the patients’ needs, as in real-life healthcare, participants did not receive standardized treatment that could be evaluated easily. Many patients received interventions that counter frailty, such as physiotherapy, advice on diet, nutritional support, and pharmacological optimization [23]. Personal factors known to influence health, such as self-efficacy, coping, and resilience, were not measured in this study. Future studies should seek to clarify which interventions are most effective in which patients, to aid the implementation of cost-effective preventive measures.

Frailty was more prevalent in this study than in other studies conducted using the CHS criteria of Fried et al., which could be explained by the selection of patients with multimorbidity and a high mean age in the present study [18, 24].

The high mortality in this study was expected, considering the high risk of mortality related to old age, multimorbidity, and frailty. We noted a tendency toward better survival in the IG in this study but after 36 months, the follow-up study found a significant difference in mortality between IG and CG, (*p* = 0.026) [14]. Possibly, the present study was underpowered due to missing data and shorter follow-up. This finding may indicate that complex interventions such as CGA take time to implement and have effect on already frail and pre-frail patients.

The inclusion criteria applied in this study has been used in studies and governmental reports on older people with multimorbidity in Sweden. Data used to identify these patients (age, diagnoses, and hospitalizations) are readily available in the medical data warehouse of Sweden’s healthcare system, and perhaps also in other countries and settings. In the present study, this definition generated a population of which 90% of participants were pre-frail or frail. Thus, this definition appears to be useful for the identification of older patients in need of CGA.

### Limitations

Some aspects of this study limit the interpretation of the results. First, it was a single-center study with a study population of older individuals with multimorbidity, which somewhat hampers generalization of the results. However, the study population is very similar to the patients at a geriatric clinic, and this real-life setting was an important factor for the success of this intervention study. Second, the amount of missing data is high but the proportions of patients that declined, had no data or had inconclusive data on frailty were similar in both groups at baseline and at 24 months. Because of this, we do not attribute the missing data as caused by the intervention itself. Some patients were too weak to perform parts of the assessments, which may have resulted in underestimation of the number of frail participants in both groups.

The original operationalization of the frailty phenotype is seldom used, as researchers adapt the measures to their own settings, with the risk of generating slightly different populations of frail individuals [24]. In this study, two CHS frailty criteria (slowness and low activity level) were modified, and weight loss at baseline was estimated by patients, rather than measured directly. Weakness and exhaustion were assessed in manner similar to those developed by Fried et al. [18]. This limitation, which is not exclusive to this study, limits the comparability of studies using the CHS definition [24]. However, it does not detract from the main findings of this study.

The CHS criteria do not address all factors described as having important effects on frailty, such as cognition and multimorbidity. This was compensated for somewhat in this study, as all participants had multimorbidity. The mean MMSE score at baseline was 26, suggesting that the study population was characterized by slightly reduced cognitive function.

## Conclusion

This study has shown that a multidisciplinary intervention in an outpatient setting had positive effects on frail older people with multimorbidity and high health care utilization. We conclude that outpatient CGA delays the progression of frailty and may contribute to the improvement of frailty status. Future studies should consider longer follow-up periods and also focus on what interventions within outpatient CGA are the most effective.
